# Interactions Between Phenolic Acids and Microorganisms in Rhizospheric Soil From Continuous Cropping of *Panax notoginseng*

**DOI:** 10.3389/fmicb.2022.791603

**Published:** 2022-02-24

**Authors:** Limei Bao, Yuyan Liu, Yafang Ding, Junjie Shang, Yunlin Wei, Yong Tan, Futing Zi

**Affiliations:** ^1^Faculty of Science, Kunming University of Science and Technology, Kunming, China; ^2^Faculty of Life Science and Technology, Kunming University of Science and Technology, Kunming, China

**Keywords:** *P. notoginseng*, continuous cropping soil sickness, microbial diversity, phenolic acids, autotoxicity

## Abstract

Large-scale intensive cultivation has made continuous cropping soil sickness more serious for *Panax notoginseng* in Yunnan. Autotoxic substances can promote the occurrence of continuous cropping soil sickness. Phenolic acids exert a strong autotoxic effect on *P. notoginseng*. Based on UPLC-MS/MS, the levels of six phenolic acids with the strongest autotoxicity of *P. notoginseng* rhizospheric soil were tested. Based on Illumina MiSeq high-throughput sequencing technology, the variation in the microbial diversity in the rhizospheric soil was used as an index to explore the interactions between phenolic acids and the soil microorganisms of the *P. notoginseng* rhizosphere. (1) Continuous *P. notoginseng* cropping significantly changed the microbial community structure. Continuous cropping increased bacterial Chao1 index and Shannon index and decreased fungal Shannon index. After *P. notoginseng* disease, bacterial Shannon index reduced and fungal Chao1 index decreased. (2) Phenolic acid significantly changed the bacterial community structure. VA significantly reduced the bacterial Shannon index. Exogenous *p*-HA, FA, SA, and VA significantly increased the fungal Chao1 index and *p*-HA showed the most significant effect. Para-HA affected bacterial specificity, and VA affected fungal specificity. (3) VA was positively correlated with most fungi and bacteria. Para-HA was positively correlated with *Lelliottia* and *Flavobacterium*. Para-HA was also positively correlated with plant pathogens (*Fusarium* and *Ilyonectria*). Para-HA and VA were able to promote the growth of primary pathogenic bacteria. Thus, *p*-HA and VA are the main phenolic acid-autotoxin substances in *P. notoginseng* under continuous cropping. (4) A correlation analysis of soil environmental factors associated with fungal and bacterial communities showed that AK, TN, OM, and HN were most strongly correlated with soil microorganisms. (5) The microorganisms in the rhizosphere of 3-year-old soil planted with *P. notoginseng* exhibited obvious effects on the degradation of the four phenolic acids. The effect of soil microorganisms on phenolic acids was first-order kinetic degradation with a high degradation rate and a half-life of less than 4.5 h. The results showed that phenolic acids could promote the growth of pathogenic bacteria. And the interaction between rhizospheric soil microorganisms and phenolic acids was the main cause of the disturbance of *P. notoginseng* rhizosphere microflora.

## Introduction

Sanqi [*Panax notoginseng* (Burkill) F. H. Chen] is a perennial medicinal plant in the genus *Panax*. The dried rhizomes of *P. notoginseng* can relieve blood stasis and stop bleeding, promote blood circulation and relieve pain. These rhizomes are primarily used to treat hemorrhagic disease, bruising injuries, blood stasis, swelling, and pain (Macdermot, 1950; Commission, 2015; Xu et al., 2018). Wenshan Prefecture in Yunnan Province is an area that is famous for the production of genuine *P. notoginseng*; it has a warm and rainy climate that is very suitable for growing *P. notoginseng*. The growth cycle of *P. notoginseng* is generally 3 years or more, and the cultivation process requires large amounts of fertilizers and pesticides, which lead to the deterioration of the physical and chemical properties of the soil, an imbalance of nutrients and microbial communities, and allelopathy and autotoxicity; collectively, these ailments are commonly known as the continuous cropping soil sickness of *P. notoginseng* (Tan et al., 2017). Due to the increasing demand for *P. notoginseng* in recent years, large-scale intensive planting has made continuous cropping soil sickness increasingly serious, which strongly affects the quality and yield of *P. notoginseng* (Yang et al., 2016; Qiao et al., 2020).

There are many factors that lead to continuous cropping soil sickness, including root exudates that induce changes in rhizospheric soil microorganisms, allelopathic interactions of soilborne pathogens, imbalances of rhizospheric microflora aggravated soil acidification, aggravation of soil fungal diseases caused by increases in rhizospheric pathogens and declines in beneficial bacteria, and the development of viral diseases (Wu and Lin, 2020). Alteration of soil microbial community structure is one of the important reasons for continuous cropping soil sickness. The numbers of microorganisms and physiological groups as well as the levels of soil enzyme activities and nutrients in soil planted in *P. notoginseng* across different planting and fallow years were studied. The structure of the soil microbial community and the soil nutrient balance were found to have changed after *P. notoginseng* cultivation. It was assumed that the changes in the biological community were not conducive to *P. notoginseng* growth (Zhang et al., 2019). Other studies have also found that an increase in the number of *P. notoginseng* planting years will cause an imbalance in the rhizospheric microbiota, increase the abundance of pathogenic fungal genera in the rhizosphere, decrease the abundance of some beneficial fungal genera, weaken the growth tendency of *P. notoginseng*, and significantly increase the incidence of disease (Tang et al., 2020). Long-term continuous cropping has been found to reduce the soil microbial diversity of the cotton (*Gossypium* spp.) rhizosphere and resulted in continuous cropping soil sickness, which led to a reduced cotton yield (Gu et al., 2012). Continuous cropping of maize (*Zea mays* Linn.) destroys the balance of the soil microflora, reducing the number of beneficial microbes and increasing populations of soil fungi, which causes serious disease and reduces the main soil nutrients (Xing et al., 2011). Continuous cucumber cropping significantly affects the abundance and community structure of soil fungi and increases the incidence of soil diseases (Li Y. et al., 2020). Therefore, changes in the rhizospheric soil microbial community can affect the growth of host plants.

Studies have shown that during the continuous cropping process, plants primarily communicate with the soil through root exudates, which can directly or indirectly change the soil microbial community, thus promoting the occurrence of continuous cropping soil sickness (Wu and Lin, 2020). For example, exudates of false starwort (*Pseudostellaria heterophylla*) roots have been found to mediate changes in soil microbial community structure and functional diversity, which affected the growth of the plants (Wu et al., 2016). Peanut (*Arachis hypogaea* Linn.) root exudates selectively inhibit or stimulate certain bacterial and fungal groups, promote changes in the soil microbial community and inhibit the growth of peanut plants (Li Y. et al., 2014). In conclusion, interactions between chemical substances secreted by plant roots and rhizospheric soil microorganisms cause changes in the rhizospheric soil microbial community, which is the primary reason for continuous cropping soil sickness (Yang et al., 2012; Dong et al., 2016; Qian et al., 2016; Tan et al., 2017; Feng et al., 2020).

Chemicals in rhizospheric soil influence microbial community composition. Allelochemicals can reduce soil microbial diversity, significantly affect the genetic structure and carbon metabolism capacity of microbial communities, and lead to imbalances in soil microbial ecosystems (Li X. et al., 2014). Some researchers have simulated the selective effects of allelochemicals secreted by plant roots on different microorganisms in the rhizosphere, and have found that root exudates can selectively inhibit the growth of beneficial microorganisms and promote the proliferation of pathogenic microorganisms in the soil (Wu et al., 2016). Metabolites of *P. notoginseng* are detected in rhizospheric soil allelochemicals and primarily include p-hydroxybenzoic acid, vanillic acid, syringic acid, p-coumaric acid, ferulic acid, and benzoic acid. Allelopathy testing has confirmed their order from strong to weak: p-coumaric acid, benzoic acid, vanillic acid, ferulic acid, p-hydroxybenzoic acid, and syringic acid (Wu et al., 2014). In a study on the influence of exogenous p-hydroxybenzoic acid on the soil microbial community of the cucumber rhizosphere, p-hydroxybenzoic acid decreased the Shannon–Wiener index of the rhizospheric bacterial community and increased the Shannon–Wiener index of the rhizospheric fungal community (Zhou et al., 2012). Different concentrations of cinnamic acid, phthalic acid, p-hydroxybenzoic acid and their mixtures have been found to decrease the quantities of peanut rhizospheric soil bacteria and actinomycetes, microbial biomass carbon and nitrogen content, and respiration intensity, increase the quantity of fungi, and promote the occurrence of soil sickness in peanut (Liu et al., 2018). Studies have also shown that phenolic acids such as gallic acid, salicylic acid, hydrocinnamic acid, and benzoic acid can increase fungal richness, change fungal community structure and increase pathogen loads (Li et al., 2018). Previous studies have confirmed that phenolic acids can significantly affect the biomass, diversity and community structure of soil microorganisms (Qu and Wang, 2008).

Several studies have shown that the accumulation of phenolic acids secreted by plant roots can mediate changes in the structure and functional diversity of soil microbial communities and indirectly affect the growth and development of host plants (Wu et al., 2016, 2017). Therefore, it has been speculated that interactions between phenolic acids secreted by *P. notoginseng* roots and rhizospheric soil microorganisms may disrupt the root microecological balance and promote the occurrence of continuous cropping soil sickness. However, there are still no reports on this topic. An increasing number of studies have shown that disturbances of the microflora mediated by root exudates play a key role in continuous cropping soil sickness (Wu et al., 2016). However, studies on continuous cropping soil sickness of *P. notoginseng* primarily focus on the isolation, identification and biological testing of allelochemicals, and the regulation of soil microorganisms by phenolic acids during *P. notoginseng* planting remains relatively unclear. On this basis, the interactions between phenolic acids secreted by *P. notoginseng* roots and soil microorganisms were studied here.

The innovative aspect of this study lies in its application of results from both field investigations and laboratory experiments to determine the interactions between phenolic acids and *P. notoginseng* rhizospheric soil microorganisms. To simulate *P. notoginseng* continuous cropping, autotoxic substances were added exogenously to regulate rhizospheric soil microorganisms. In addition, microorganisms exhibit detoxification functions and can degrade autotoxins in soil. This study systematically and comprehensively revealed the interactions between autotoxins and the rhizospheric soil microbial-plant cycle. Here, healthy and diseased rhizospheric soils under *P. notoginseng* cultivation for different numbers of years in Wenshan Prefecture, Yunnan Province were selected. UPLC-MS/MS and Illumina MiSeq high-throughput sequencing technology were used to determine the contents of benzoic acid (BA), p-hydroxybenzoic acid (*p*-HA), ferulic acid (FA), syringic acid (SA), vanillic acid (VA), and p-coumaric acid (*p*-CA) and microbial community structure in different soils (Wu et al., 2014). Interactions between phenolic acid autotoxic substances and soil microorganisms from the *P. notoginseng* root system were explored to provide a basis for solving these obstacles to continuous cropping. The study was designed to test these hypotheses: (1) The concentration of phenolic acids in *P. notoginseng* rhizospheric soil is related to the health status and number of years of *P. notoginseng* planting. (2) Continuous cropping could significantly change the composition of the soil microbial community in the *P. notoginseng* rhizosphere. (3) Exogenous phenolic acids can change the composition of the soil microbial community. Phenolic acids are strongly associated with soil microorganisms and can promote the growth of plant pathogens. (4) Soil microorganisms can degrade phenolic acids and rapidly decrease their concentrations. Finally, the interaction between soil microorganisms and phenolic acids reached a dynamic balance. If balance is disturbed, disease can occur in host plants.

## Materials and Methods

### Site Description and Soil Sampling

In this study, rhizospheric soil planted with *P. notoginseng* for different numbers of years was collected to determine the phenolic acid contents of the soil. We analyzed the physical and chemical properties of soil samples and explored the interactions between phenolic acids and rhizospheric soil microorganisms. Soil samples were collected in Wenshan Prefecture, Yunnan Province (103.58′ N, 23.87′ E) in November 2020, and the sampling site was located near Pingyuan Street, Yanshan County. This area has a subtropical continental monsoon climate, with an annual average temperature of 25°C and an annual precipitation of 1,308 mm.

At the *P. notoginseng* cultivation site, control soil (CK), 1-year-old healthy rhizospheric soil (1Y), 2-year-old healthy rhizospheric soil (H-2Y), 2-year-old diseased rhizospheric soil (S-2Y), 3-year-old healthy rhizospheric soil (H-3Y), and 3-year-old diseased rhizospheric soil (S-3Y) were collected. Soil collected under the same conditions from 3 to 5 cm below the surface layer where nothing was planted was used as the control soil. Healthy rhizospheric soil refers to the soil attached to the root tubers of *P. notoginseng* plants that have been continuously planted for 2 or 3 years without disease or insect pests. The diseased soil was rhizospheric soil that from plants showing root rot from the same experimental plot (Tan et al., 2017). In this study, a total of six types of soils (CK, 1Y, H-2Y, S-2Y, H-3Y, and S-3Y) were collected. Three greenhouses were selected for each soil (the soil in each greenhouse was managed in the same way) for repeated collection using the five-point sampling method. The soil attached to the rhizosphere of *P. notoginseng* plants was shaken off into self-sealed bags, marked and brought back to the laboratory (Riley and Barber, 1970). Three biological replicates were performed for each type of soil sample. There were a total of 18 replicates among 6 types of soil sample. A portion of the soil was used for physicochemical property analysis and phenolic acid degradation experiments, while the remainder was stored at −80°C for subsequent soil microbial community diversity analysis.

### Soil Physical and Chemical Properties

Control soil (CK), 1-year-old healthy rhizospheric soil (1Y), 2-year-old healthy (H-2Y) and diseased rhizospheric soil (S-2Y), and 3-year healthy (H-3Y) and diseased rhizospheric soil (S-3Y) were selected, and analysis of each sample was repeated five times to determine its physical and chemical properties. Soil pH was measured in a 1:2.5 soil-water (W/V) suspension with a pH 700 pH meter (United States, Uutech) (Qiu et al., 2012). The carbon dioxide content was quantitatively determined by potassium dichromate oxidation and external heating methods, and the organic matter (OM) content of each sample was calculated by carbon nitridation (Nelson and Sommers, 1996). The total nitrogen (TN) was determined using an elemental analyzer (Vario EL III, Germany) (Bremner, 1960). After each sample was infused with sodium hydroxide, the frit was dissolved in water and dilute sulfuric acid, the anti-chromogenic agents molybdenum and antimony were added to the solution, and the total phosphorus (TP) content in the soil was quantitatively determined with an ultraviolet spectrophotometer (L5) (GB/T9837-1988). The soil organic matter was removed by heating and oxidation with nitric acid and perchloric acid. After the silicate was digested with hydrofluoric acid, the remaining residue was dissolved with hydrochloric acid, and the total potassium (TK) in the soil was determined using a ZEENIT700P graphite furnace atomic absorption spectrometer (GB/T9836-1988). The hydrolytic nitrogen (HN) in the soil was determined by the alkaline hydrolysis diffusion method (Shen et al., 2011). The available phosphorus (AP) in the soil was extracted with sodium bicarbonate, and its content was determined by the molybdenum antimony anti-colorimetric method (Gyaneshwar et al., 2002). Extraction was performed with neutral ammonium acetate solution and soil available potassium (AK) was determined by flame photometry (Mclean and Watson, 1985). In this study, each sample was analyzed three times.

### Determination of the Phenolic Acids Concentration in the Soil

Three g each of the control soil (CK), 1-year-old healthy rhizospheric soil (1Y), 2-year-old healthy rhizospheric soil (H-2Y), 2-year-old diseased rhizospheric soil (S-2Y), 3-year-old healthy rhizospheric soil (H-3Y), and 3-year-old diseased rhizospheric soil (S-3Y) were weighed and placed in separate 50 ml centrifuge tubes; then, 20 ml of chromatographic methanol was added to each tube, and phenolic acids were extracted ultrasonically for 40 min and centrifuged at 4,500 r/min for 10 min. After centrifugation, the residue remaining in each tube was incubated with 20 ml of methanol for ultrasonic extraction for 40 min and centrifugation at 4,500 r/min for 10 min (Dong et al., 2018). Each supernatant was combined twice and concentrated by rotary evaporation in a water bath at a temperature of 50°C until it was solvent-free. Then, 5 ml of methanol was added to each dried rotary steaming flask, and the solution was transferred to a 10 ml round-bottom flask after all the substances were dissolved. Each solution was concentrated to dry by rotary steaming (Zhong et al., 2020). The HLB columns were each washed with 2 ml methanol and then activated with 2 ml 1 M formic acid. Two milliliters of each extract (in a methanol:water:formic acid volumetric ratio of 15:4:1) was added to a rotary steaming flask, the supernatant was absorbed through the HLB column, and the eluent was collected. The HLB column was rinsed with 1 ml of extract, and the eluent was collected. The two eluents from each sample were combined for steam drying; then, 200 μl of extract was added and filtered through a 0.22 μm micron filter membrane. Quantitative analysis was performed using an UPLC-MS/MS (Tsushima 8050) in MRM mode (Jia et al., 2020). Each sample was analyzed three times.

### Degradation of Phenolic Acids by Rhizospheric Soil Microorganisms

Generally, two and 3-year-old *P. notoginseng* showed the highest incidence of root rot. *P. notoginseng* planted in the field is generally harvested in 3 years. Considering the number of continuous cropping years and field practices used in *P. notoginseng* cultivation, 3-year-old healthy *P. notoginseng* rhizospheric soil was selected for the degradation experiment. Six samples of 36 g of 3-year-old healthy rhizospheric soil from around *P. notoginseng* plants was measured and placed in separate 250 ml tapered flasks with stoppers. Maximum values from results of the field investigation in this study and previous reports were adopted for the experimental setting. BA (5 μg/g), *p*-HA (5 μg/g), FA (5 μg/g), SA (5 μg/g), VA (10 μg/g), and *p*-CA (30 μg/g) (Wu et al., 2014) were each added to a separate conical flask containing a healthy soil sample and incubated in a thermostatic chamber at 25°C, and the conical flasks were aerated for 1 hour every 24 h. The soil moisture content was maintained at 70%. Three g of soil was randomly weighed from each conical flask at 0, 6, 12, 24, 48, and 72 h (Guo et al., 2011). The concentration of phenolic acids in each soil sample was quantitatively analyzed according to the first-order kinetic equations LnC(t)=*LnC*(t_0_)-Kt and t_1/2_=*Ln2*/K. The half-life of each autotoxic substance (t_1/2_) was calculated, where C is the concentration, T is the time, and K is the degradation rate (Xia et al., 2015). The method for determining soil phenolic acid concentrations was the same as that described above. Each phenolic acid sample was examined five times to explore the degradation ability of the soil microorganisms.

Total soil DNA was extracted from the rhizospheric soil 72 h after culture from 5 replicates and sequenced for analysis (Luo et al., 2020). The effects of the six phenolic acids on the soil microorganisms of *P. notoginseng* rhizospheres were studied.

### DNA Extraction, PCR Amplification and Illumina MiSeq Sequencing

Total DNA from the soil microorganisms was extracted by using the DNeasy PowerSoil^®^ Kit from QIAGEN, Germany. Three parallel samples were prepared for each sample and amplified by PCR (instrument model ETC811, Dongsheng Scientific Instruments Co., Ltd.). The diluted genomic DNA was used as a template and amplified with specific primers containing barcodes according to the selected sequencing region. The amplified products were subjected to gel electrophoresis, recovered and quantified using a QuantiFluor™ fluorometer. The bacterial genome (16S rDNA V3-V4 gene region) primers were 341F: CCTACGGGNGGCWGCAG and 806R: GGACTACHVGGGTA TCTAAT (Neefs et al., 1991). The fungal genome (ITS2 gene region) primers were ITS3-KYO2: GATGAAGAACGYA GYRAA and ITS4: TCCTCCGCTTATTGATATGC (Toju et al., 2012). Water was used as a negative control for the PCR procedure in each sample, and no bands were amplified from the negative controls. The purified amplification products were mixed in equal amounts, a sequencing connector was added to construct the sequencing libraries, and the Illumina PE250 library was sequenced on the machine. To ensure the reliability and validity of the data, FASTP was used to filter reads from the original dataset generated by the Illumina MiSeq platform. FASTP filtered out low-quality reads: (1) reads with over 10% of nucleotides (N) unknown were removed, and (2) reads for which less than 50% of bases showed quality scores (Q-values) > 20 were removed. FLASH was used to splice double-ended reads into tags and then filter low-quality tags to obtain clean tags (Magoc and Salzberg, 2011; Chen et al., 2018). The clean tags were clustered into operational taxonomic units (OTUs) of ≥ 97% similarity using the UPARSE (version 9.2.64) pipeline. All chimeric tags were removed using the UCHIME algorithm, and effective tags were finally obtained for further analysis. The tag sequence with the highest abundance was selected as the representative sequence within each cluster. All singleton sequencing data were removed from the sequencing data. OTUs were obtained by clustering with 97% sequence similarity (Table 1). The sequencing data were compared with SILVA (16S) and UNITE (ITS) database to obtain biotaxonomic information.

**TABLE 1:** Sequencing results for bacteria and fungi in *P. notoginseng* rhizospheric soil.

	Bacterial			Fungal		
Sample	Total tags	Singleton Tags	OTUs (97%)	Total tags	Singleton tags	OTUs (97%)
CK1	122,090 ± 1,483	32,388 ± 2,333	1,811 ± 66	122,020 ± 398	3,075 ± 546	824 ± 58
H-1Y	121,917 ± 927	45,622 ± 242	3,530 ± 50	125,520 ± 2,294	1,413 ± 66	725 ± 15
H-2Y	125,494 ± 1,117	47,534 ± 813	3,809 ± 92	122,314 ± 617	1,424 ± 64	695 ± 12
S-2Y	119,990 ± 1,915	45,445 ± 2,218	3,078 ± 300	124,710 ± 1,758	1,248 ± 45	519 ± 29
H-3Y	120,785 ± 1,895	42,887 ± 1,086	3,303 ± 77	118,181 ± 1,842	1,378 ± 98	551 ± 11
S-3Y	120,375 ± 1,558	44,317 ± 2,051	2,899 ± 91	126,227 ± 153	1,330 ± 72	476 ± 21
p-HA	120,266 ± 796	40,944 ± 239	3,585 ± 9	125,993 ± 711	1,628 ± 41	685 ± 4
BA	118,815 ± 2,128	38,380 ± 305	3,564 ± 12	123,282 ± 1,113	1,399 ± 37	580 ± 4
SA	121,742 ± 759	37,141 ± 1,214	3,594 ± 4	121,942 ± 2,507	1,366 ± 9	624 ± 9
FA	120,656 ± 1,641	33,976 ± 836	3,536 ± 20	124,772 ± 1,619	1,372 ± 49	558 ± 9
VA	120,634 ± 702	41,851 ± 2,229	3,368 ± 45	126,420 ± 868	1,316 ± 95	609 ± 2
p-CA	121,848 ± 885	35,783 ± 569	3,551 ± 9	122,660 ± 1,245	1,311 ± 57	510 ± 9
CK2	118,783 ± 920	31,884 ± 1,602	3,602 ± 23	119,305 ± 1,208	1,116 ± 115	500 ± 13

### Data Analysis

We used QIIME software for alpha diversity index analysis (Kuczynski et al., 2012). Based on the intersample distance index, PCoA analysis was performed using the R language vegan package (Liu and Li, 2021). The degree and direction (positive or negative) of correlation between each environmental factor and each species were evaluated by Pearson analysis. FUNGuild was used to annotate fungal functions based on OTU (97%) abundance table information.

SPSS 21.0 software (SPSS Inc., United States) was used to analyze the data, and the homogeneity of variance was tested before the statistical analysis. Statistical analysis was performed using a one-way analysis of variance (ANOVA) and Duncan’s multi-interval test (*P* < 0.05). Sequencing data were provided by Guangzhou Gene Denovo Biotechnology Co., Ltd. Drawings and data analysis were completed by the first author of this study. Raw sequences were uploaded to the NCBI BioProject database (Accession: PRJNA757903).

## Results

### Analysis of Soil Physical and Chemical Properties

In the analysis of soil physical and chemical properties, the pH of the rhizospheric soil from planted *P. notoginseng* ranged from 5.18 to 6.29. The pH of the S-2Y rhizospheric soil around *P. notoginseng* plants was significantly higher than that of the control soil. Values of OM, TN, HN, and AP in healthy rhizospheric soil were significantly higher than those in the control soil. All the physical and chemical indexes of sick rhizospheric soil except pH were significantly higher than those of the control group. The AK content of healthy soil was significantly higher than that in sick rhizospheric soil (Table 2).

**TABLE 2:** Physical and chemical properties of different cultivated soils.

Sample	pH	OM (g/kg)	TN (g/kg)	TP (g/kg)	TK (g/kg)	HN (mg/kg)	AP (mg/kg)	AK (mg/kg)
CK	5.26 ± 0.63b	14.24 ± 3.46c	0.82 ± 0.11c	0.64 ± 0.19d	4.52 ± 0.21b	65.63 ± 10.20b	4.55 ± 3.49c	107.67 ± 52.64d
1Y	5.72 ± 0.01ab	27.72 ± 0.22a	1.38 ± 0.02ab	2.07 ± 0.09a	5.30 ± 0.34a	119.69 ± 3.81a	88.50 ± 3.49a	422.35 ± 14.08b
H-2Y	5.78 ± 0.53ab	24.95 ± 0.99ab	1.30 ± 0.08ab	0.94 ± 0.05cd	4.72 ± 0.16ab	108.46 ± 5.63a	24.26 ± 5.91b	219.10 ± 31.22cd
H-3Y	5.18 ± 0.13b	23.82 ± 1.08ab	1.24 ± 0.05b	1.57 ± 0.26b	4.76 ± 0.20ab	108.46 ± 7.86a	38.20 ± 12.6b	157.33 ± 27.53cd
S-2Y	6.29 ± 0.3a	26.02 ± 0.14ab	1.41 ± 0.02a	1.17 ± 0.14bc	5.19 ± 0.21a	120.21 ± 5.92a	29.49 ± 7.37b	614.30 ± 124.52a
S-3Y	5.18 ± 0.04b	23.48 ± 1.78b	1.25 ± 0.09ab	1.22 ± 0.26bc	5.08 ± 0.40ab	113.30 ± 20.80a	39.28 ± 12.45b	261.87 ± 58.39c

*The pH, OM, organic matter; TN, total nitrogen; TP, total phosphorus; TK, total potassium; HN, hydrolytic nitrogen; AP, available phosphorus; AK, available potassium, concentrations in P. notoginseng rhizospheric soil varying in number of planting years are shown as the means ± SE. Columns with different letters are significantly different (LSD-test, p < 0.05).*

### Determination and Analysis of Phenolic Acids in Rhizospheric Soil

**FIGURE 1:**
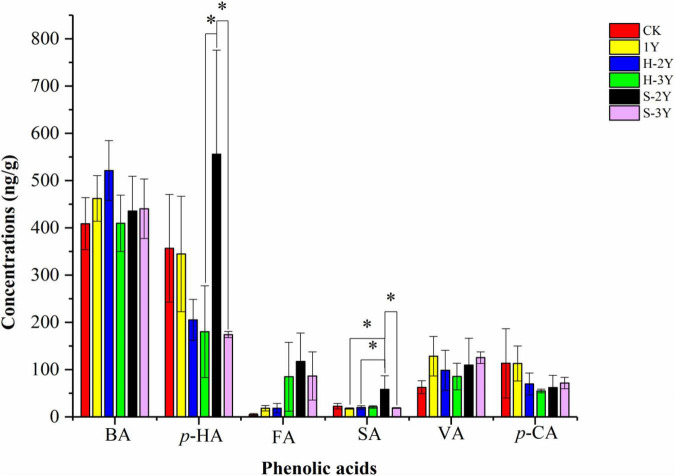
Phenolic acid concentrations. Red represents control soil, yellow represents 1-year-old *P. notoginseng* rhizospheric soil (H-1Y), blue represents 2-year-old healthy rhizospheric soil (H-2Y), green represents 3-year-old healthy rhizospheric soil (H-3Y), black represents 2-year-old diseased rhizospheric soil (S-2Y), and purple represents 3-year-old diseased rhizospheric soil (S-3Y). BA is benzoic acid, *p*-HA is p-hydroxybenzoic acid, FA is ferulic acid, SA is syringic acid, VA is vanillic acid, and *p*-CA is p-coumaric acid; * indicates a significant difference LSD-test, (*p* < 0.05).

The phenolic acids detected in *P. notoginseng* rhizospheric soil primarily included benzoic acid (BA), p-hydroxybenzoic acid (*p*-HA), ferulic acid (FA), syringic acid (SA), vanillic acid (VA), and p-coumaric acid (*p*-CA). The concentration of *p*-HA in the 2-year-old diseased rhizospheric soil (S-2Y) of *P. notoginseng* was significantly higher than that of the 3 years of rhizospheric soil (H-3Y and S-3Y). The concentration of SA in the 2-year-old diseased rhizospheric soil (S-2Y) of *P. notoginseng* was significantly higher than that of 1-year-old healthy rhizospheric soil (1Y), 2-year-old healthy rhizospheric soil (H-2Y) and 3-year-old diseased rhizospheric soil (S-3Y). The concentrations of *p*-HA (555.66 ± 121.95), FA (117.34 ± 9.86), and SA (58.28 ± 1.40) were highest in S-2Y among all the measured samples (Figure 1).

### Alpha Diversity Analysis

**FIGURE 2:**
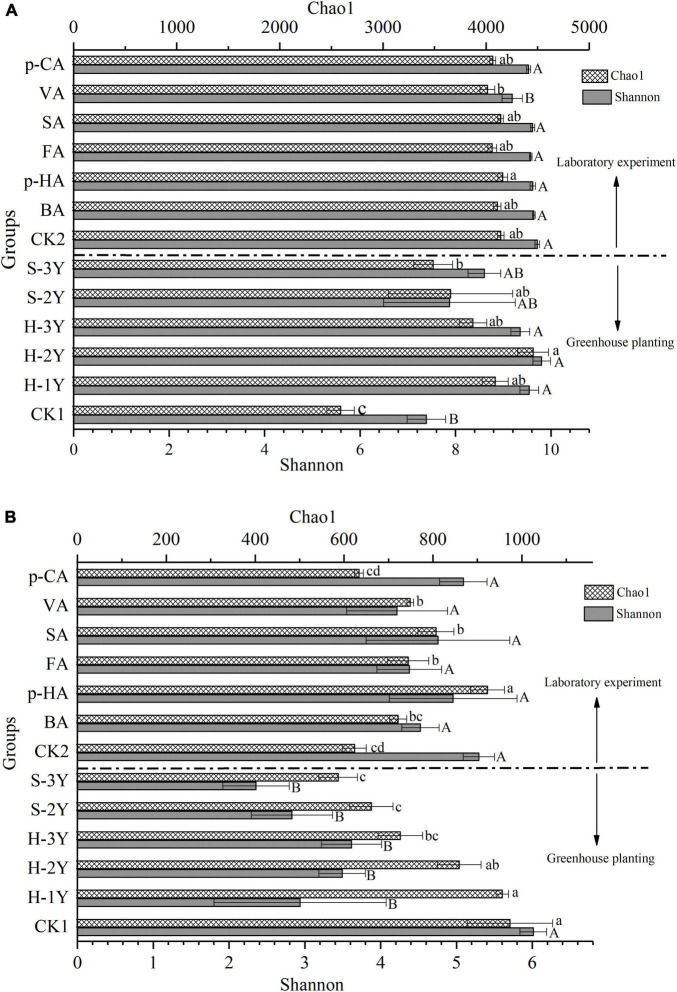
Changes in the rhizosphere soil microbial community associated with *P. notoginseng*. The bacterial Chao1 index and Shannon index **(A)** and fungal Chao1 index and Shannon index **(B)** are represented as the means ± SE (*n* = 3). CK1 is natural soil that has not been planted with *P. notoginseng*, and CK2 is the 3-year-old healthy rhizospheric soil from *P. notoginseng* cultivated for 72 h without exogenous phenolic acid addition. Different letters indicate significant differences (*P* < 0.05). Lowercase letters indicate the significant differences of Chao1 index, and uppercase letters indicate the significant differences of Shannon index. Above the dotted line are data from the indoor experiment, and below the dotted line are data from the greenhouse planting experiment.

During the greenhouse experiment, bacterial Chao1 index in the rhizospheric soil from planted *P. notoginseng* were significantly higher than values for the control soil The Shannon index of bacteria in healthy *P. notoginseng* rhizosphere soil was significantly higher than that in control group. The Chao1 index of 3-year-old diseased *P. notoginseng* rhizospheric soil (S-3Y) was significantly lower than that of 2-year-old healthy *P. notoginseng* rhizospheric soil (H-2Y). The difference between Chao1 index and Shannon index may be caused by the uneven distribution of species. All the data indicated that continuous cropping led to an overall increase in bacterial Chao1 index and Shannon index, and the occurrence of disease reduced bacterial Shannon index. The exogenous addition of VA in the laboratory significantly reduced the bacterial Shannon index (Figure 2A).

Compared with the control soil, the rhizospheric soil planted with *P. notoginseng* significantly reduced fungal Shannon index in the greenhouse experiment. The fungal Chao1 index of diseased rhizospheric soil (S-2Y and S-3Y) was significantly lower than that of healthy rhizospheric soil (H-2Y and H-1Y) and control soil. The result showed that the fungal Chao1 index was significantly decreased after *P. notoginseng* disease. The Chao1 index for fungi in the rhizospheric soil decreased with increasing number of cultivation years, the 3-year-old healthy rhizosphere soil (H-3Y) decreased most significantly. Exogenous *p*-HA, FA, SA, and VA significantly increased the Chao1 index of the fungi compared with the control soil, and *p*-HA showed the most significant effect. Exogenous phenolic acid had no effect on Shannon index of fungal community, but significantly increased Chao1 index. The results indicated that the addition of exogenous phenolic acid could significantly improve the category richness of fungal species, but did not have a good effect on the evenness. Therefore, exogenous phenolic acids did not obvious change the fungal Shannon index (Figure 2B).

### Beta Diversity Analysis

**FIGURE 3:**
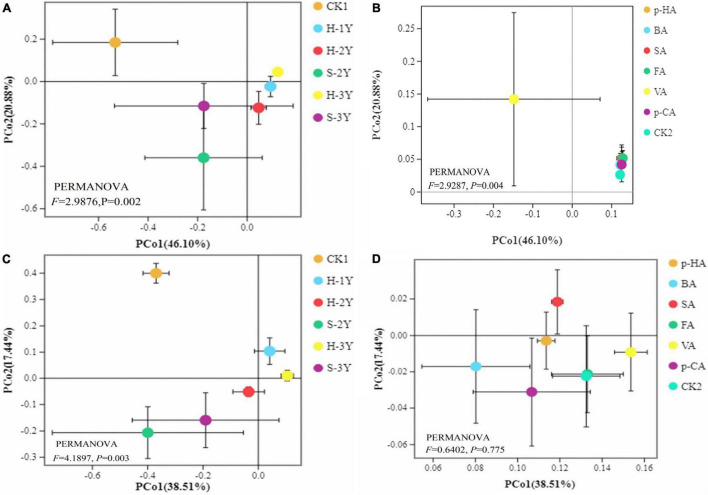
Principal co-ordinate analysis (PCoA). Greenhouse bacterial groups **(A)**, laboratory bacterial group **(B)**, greenhouse fungal groups **(C)**, laboratory fungal group **(D)**. Each point represents a sample. The closer the points are to each other on the plane, the more similar the bacterial community structure of the samples.

The continuous cropping of *P. notoginseng* significantly [by permutational multivariate analysis of variance (PERMANOVA), *P* < 0.05] altered the community structure of the soil bacteria and fungi (Figures 3A,B). Exogenous phenolic acids significantly (PERMANOVA, *P* < 0.05) changed the soil bacterial community structure but had no significant (PERMANOVA, *P* > 0.05) effect on the fungi (Figures 3C,D).

### Microbiological Specificity Analysis

The specificity of microorganisms in rhizospheric soil decreased after *P. notoginseng* was planted. The species specificity of diseased *P. notoginseng* rhizosphere soil was lower than that of healthy soil and control soil (Supplementary Tables 1, 2). Exogenously added phenolic acids changed the species specificity. There were 13 kinds of specific bacteria in VA treatment group, which was much higher than that in control group CK2 (Supplementary Table 1). There were 22 specific fungi in *p*-HA treatment group, fungal species specificity was increased compared with the control group. The result indicated that *p*-HA could improve the species specificity of fungi (Supplementary Table 2).

### Characteristics of the Rhizospheric Soil Microbial Communities and the Effects of Phenolic Acids

**FIGURE 4:**
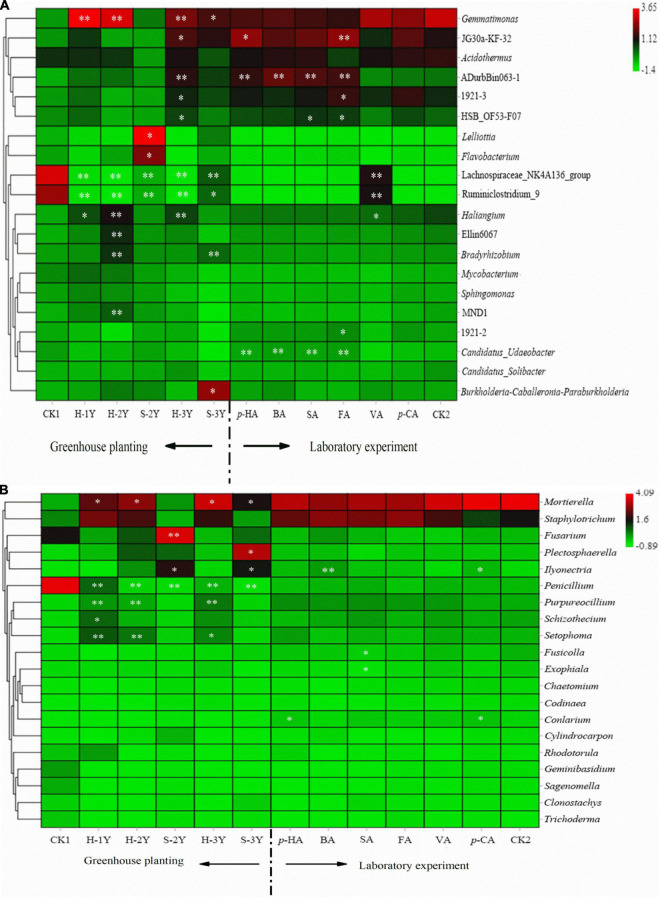
Heat map of bacterial abundances in *P. notoginseng* rhizospheric soil varying in number of planting years and treated with exogenous phenolic acids **(A)**. Heat map of fungal abundances in *P. notoginseng* rhizospheric soil varying in number of planting years and treated with exogenous phenolic acids **(B)**. Column normalization was performed for both graphs **(A,B)**. The bacterial sequencing data were compared with SILVA database to obtain bacterial taxa. Fungal sequencing data were compared with UNITE database to obtain fungal taxa. We selected the top 20 species with relative abundance above 0.1% in at least one sample for heat map analysis. CK1 is the natural soil that has not been planted with *P. notoginseng*, and CK2 is the healthy 3-year-old rhizospheric soil without exogenous phenolic acid addition. The closer the color is to red, the higher the microbial abundance. And the closer the color is to green, the lower the microbial abundance. [* indicates a significant difference from the control group,* indicates *P* < 0.05; ^**^ indicates *P* < 0.01 (LSD-test)].

The abundance of microorganisms in the rhizospheric soil was closely related to the number of years of *P. notoginseng* cultivation. The abundance of *Gemmatimonas* and *Haliangium* significant increased, while Lachnospirae-NK4a136-Group and *Ruminiclostridium*-9 significant decreased in continuously cropped healthy *P. notoginseng* rhizospheric soil. In rhizospheric sick soil of 2-year-old (S-2Y), the abundance of *Lelliottia* and *Flavobacterium* increased significantly (Figure 4A). *Flavobacterium* is a specific species in the rhizosphere soil of healthy 3-year-old *P. notoginseng* (Supplementary Table 1). In rhizospheric sick soil of 3-year-old (S-3Y) the abundance of *Gemmatimonas* increased significantly compared with control soil (Figure 4A). The interaction between allelopathic autotoxins and root microorganisms is the main cause of continuous cropping obstacles (Feng et al., 2020). In terms of bacterial community structure, VA significantly increased the abundance of *Ruminiclostridium*-9 and Lachnospirae-NK4A136-Group and significantly reduced the abundance of *Haliangium*. Para-HA, BA, FA, SA significantly increased the abundance of ADurbBin063-1 and *Candidatus-Udaeobacter* (Figure 4A).

**FIGURE 5:**
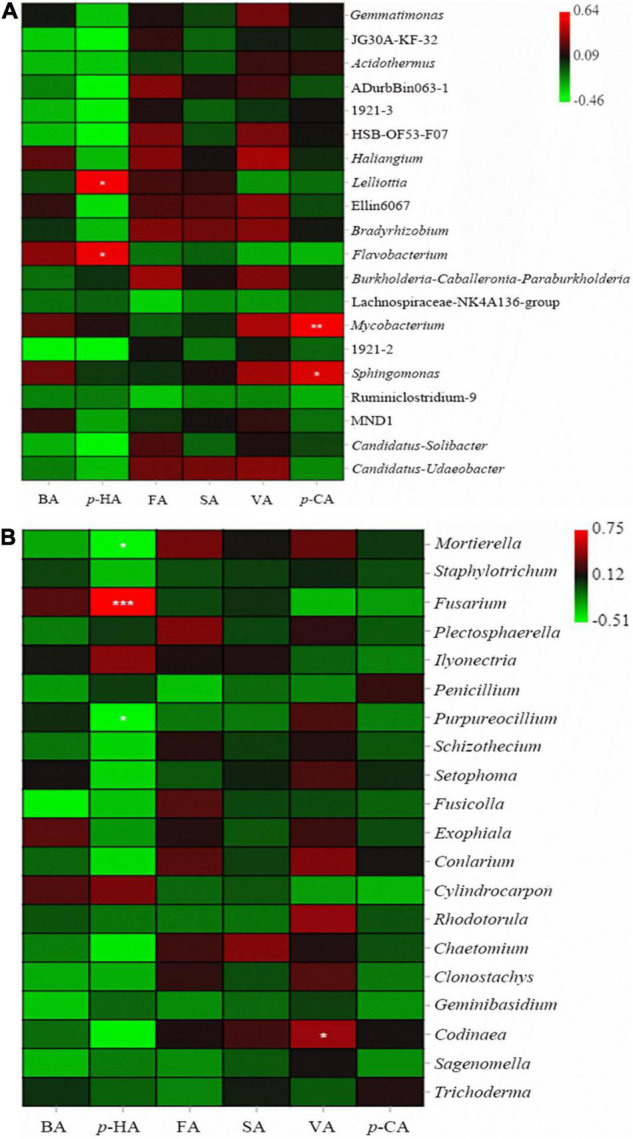
Correlations of phenolic acids with bacteria **(A)** and fungi **(B)**. The colors in the figure indicate the strength of the correlation. The closer the color is to red, the stronger the positive correlation, while the closer the color is to green, the stronger the negative correlation. * indicates a significant correlation [* indicates *P* < 0.05; ** indicates *P* < 0.01; and *** indicates *P* < 0.001 (Tukey’s test)].

Continuous cropping in healthy *P. notoginseng* caused the abundance of *Mortierella*, *Purpureocillium*, and *Setophoma* increased significantly, while *Penicillium* decreased significantly. After *P. notoginseng* was infected, the abundance of *Ilyonectria* increased significantly. In rhizospheric sick soil of 2-year-old (S-2Y), the abundance of *Fusarium* increased significantly. In rhizospheric sick soil of 2-year-old (S-3Y), the abundance of *Plectosphaerella* increased significantly (Figure 5B). Para-HA and VA significant increased the abundance of *Ilyonectria*. Para-HA and *p*-CA significantly increased the abundance of *Conlarium*. SA significantly reduced the abundance of *Exophiala* and *Fusicolla* (Figure 4B).

## FUNGuild Analysis of Fungi

FUNGuild predicted the pathogenicity of 15 species in the top 20 fungi. The genera *Fusarium*, *Ilyonectria*, *Cylindrocarpon*, and *Rhodotorula* are predicted to be plant pathogens (Table 3).

**TABLE 3:** Fungi FUNGuild.

Taxon	Guild
*Mortierella*	Endophyte-Litter Saprotroph-Soil Saprotroph-Undefined Saprotroph
*Fusarium*	Animal Pathogen-Endophyte-Lichen Parasite-Plant Pathogen-Soil Saprotroph-Wood Saprotroph
*Staphylotrichum*	Undefined Saprotroph
*Ilyonectria*	Plant Pathogen
*Plectosphaerella*	Endophyte
*Purpureocillium*	Fungal Parasite
*Mortierella*	Endophyte-Litter Saprotroph-Soil Saprotroph-Undefined Saprotroph
*Cylindrocarpon*	Plant Pathogen
*Rhodotorula*	Animal Endosymbiont-Animal Pathogen-Endophyte-Plant Pathogen-Undefined Saprotroph
*Geminibasidium*	Undefined Saprotroph
*Conlarium*	Undefined Saprotroph
*Penicillium*	Endophyte
*Sagenomella*	Undefined Saprotroph
*Exophiala*	Animal Pathogen

### Analysis of Correlations of Phenolic Acids and Rhizospheric Soil Microorganisms

There was an obvious correlation between phenolic acids and microbial abundance in *P. notoginseng* rhizospheric soil. An analysis of correlations between phenolic acids and bacteria showed that *p*-HA was significantly positively correlated with *Lelliottia* and *Flavobacterium*. Para-CA was significantly positively correlated with *Mycobacterium* and *Sphingomonas*. FA and VA were positively correlated with most bacteria (Figure 5A).

The analysis of correlations between phenolic acids and fungi showed that VA was positively correlated with most fungi, and the other 5 phenolic acids were negatively correlated with most fungi. VA was significantly positively correlated with *Codinaea*. Para-HA was significantly positively correlated with *Fusarium* and was significantly negatively correlated with *Mortierella* and *Purpureocillium*. Para-HA, BA, FA, and SA were positively correlated with *Ilyonectria* (Figure 5B).

### Degradation of Phenolic Acids by Soil Microorganisms

**FIGURE 6:**
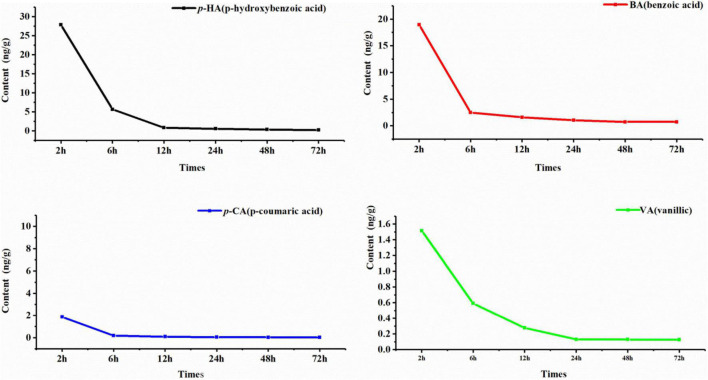
Kinetic degradation curve of phenolic acids. The horizontal column represents the degradation time, and the vertical axis represents the phenolic acid content. The black curve is *p*-HA, the red curve is BA, the blue curve is *p*-CA, and the green curve is VA.

Six phenolic acids were degraded in total, but the degradation effects of FA and SA were not obvious, so only the degradation curves of *p*-HA, BA, *p*-CA, and VA are shown. The degradation of the four phenolic acids by rhizospheric soil microorganisms was clear; degradation was rapid during the first 6 h and tended to slow after 6 h (Figure 6). According to the first-order kinetic equation, the half-lives of *p*-HA, BA, *p*-CA, and VA were 2.60, 2.05, 2.28, and 4.41 h, respectively. It follows that the rate of degradation of BA by soil microorganisms was the fastest, and that of VA was the slowest.

### Analysis of Correlations of Soil Environmental Factors and Bacterial/Fungal Communities

**FIGURE 7:**
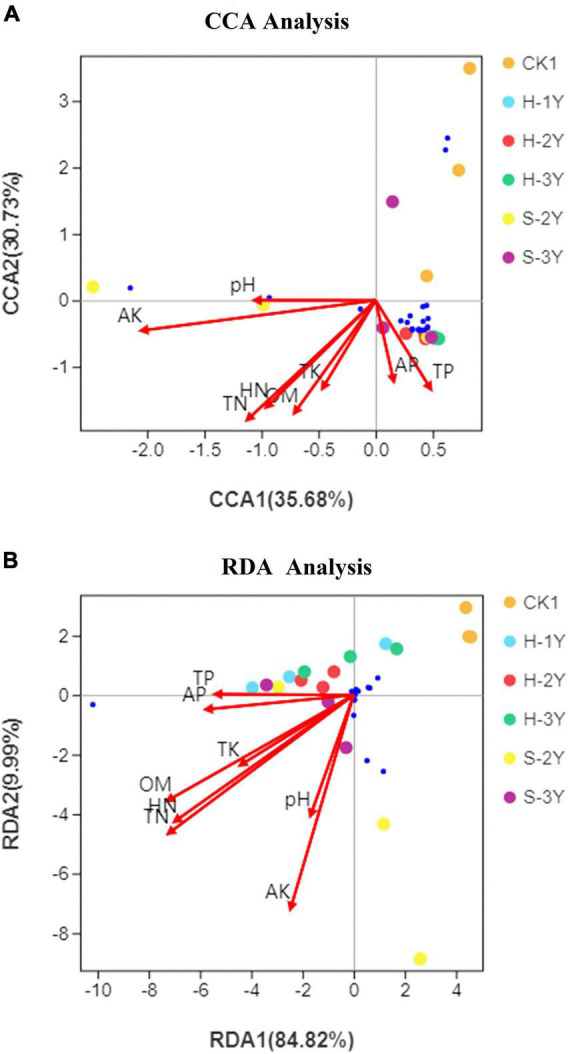
Canonical correspondence analysis of the bacterial community with soil environmental factors **(A)** and redundancy analysis of soil environmental factors with the fungal community **(B)**. OM is organic matter, TN is total nitrogen, TP is total phosphorus, TK is total potassium, HN is hydrolysable nitrogen, AP is available phosphorus, and AK is available potassium. The top 20 dominant species, whose genus names shown in the heat map Figure 4, were selected for the mapping analysis. The blue dots represent these micro flora.

According to the results of detrended correspondence analysis (DCA), the response of the bacterial community to soil environmental factors was unimodal, while the response of the fungal community was linear. Bacterial communities were examined by canonical correspondence analysis (CCA), and fungal communities were studied by redundancy analysis (RDA). The top 20 dominant species, whose genus names shown in the heat map Figure 4, were selected for the mapping analysis. The blue dots represent these micro flora. AK, TN, HN, and OM exhibited the greatest influence on the bacterial and fungal community (Figures 7A,B). The projection of species on the arrow indicates a positive correlation between abundance of species and environmental factors, while the projection on the backward extension of the arrow indicates a negative correlation. Therefore, TP and AP were positively correlated with the most bacteria. The correlation between soil environmental factors and most fungi was not obvious. In conclusion, AK, TN, HN, and OM exert the greatest influence on rhizospheric soil microorganisms.

## Discussion

### Soil Physical and Chemical Properties Are Related to Microbial Community Structure

The massive use of mineral fertilizers, especially under intensive cultivation systems, appears to exacerbate declines in soil quality by inducing acidification and salinization (Cesarano et al., 2017). Although the degradation of soil quality does not directly cause soil disease in plants, the composition and abundance of soil microorganisms are controlled by soil properties (Paterson et al., 2007). The effects of AK, OM, TN, and HN on the bacterial community were the greatest. Studies have shown that total nitrogen and organic matter had significant effects on bacterial community (Li C. et al., 2020) and the excessive application of nitrogen potash will cause soil salt accumulation and decrease the C/N ratio, resulting in changes to the plant rhizospheric soil bacterial community (He et al., 2017). Also AK, OM, TN, and HN showed the greatest effects on the fungal community. Previous studies showed that the most important factors affecting soil fungal community were organic carbon, available nitrogen, total nitrogen, and C/N ratio (Zhao et al., 2021). This conclusion is similar to the result of this study. Soil microorganisms can sense changes in soil properties caused by nitrogen fertilizer and respond in different ways (Zhao et al., 2014). Nitrogen fertilizer application has been found to reduce the abundances of microorganisms in the rhizospheric soil of plants and reduced the diversity and richness of the community (Shen et al., 2021). In conclusion, soil physical and chemical properties play an important role in the assembly of microbial communities (Tayyab et al., 2019).

### Phenolic Acids Associated With Soil Sickness

There are many factors affecting continuous cropping obstacles, such as the accumulation of soil-borne pathogens and parasites, changes in soil microbial community composition, and root exudates (Cesarano et al., 2017). Allelopathic autotoxic substances in the root exudates of *P. notoginseng* can affect plants by destroying the plant cell structure, affecting plant photosynthesis, and promoting the occurrence of continuous cropping soil sickness (Zhang et al., 2018). In particular, phenolic acids secreted by plant roots can inhibit the germination of seeds and reduce the immunity and stress resistance of plants (Feng et al., 2020). Phenolic acid autotoxins can affect plant growth by inhibiting photosynthesis. The literature indicates that ferulic acid can reduce the chlorophyll content in *P. notoginseng* seedlings, reduce the photosynthetic rate and inhibit plant growth (Shen et al., 2016). The FA concentration increased with increasing number of *P. notoginseng* planting years, and reached a maximum value for S-2Y. Para-HA and SA acid contents were highest for S-2Y. Among the collected samples, the S-2Y rhizospheric soil sickness was the most serious. Therefore, FA, *p*-HA, and SA may be related to soil sickness in *P. notoginseng*.

### Effects of *Panax notoginseng* Continuous Cropping on Soil Microorganisms

Long-term continuous cropping changes the soil microbial diversity (Sun et al., 2014; Zhao et al., 2014). *P. notoginseng* continuous cropping increased bacterial Chao1 index and Shannon index and decreased fungal Shannon index. After *P. notoginseng* disease, bacterial Shannon index reduced and fungal Chao1 index increased. Beta-diversity analysis also showed that continuous cropping significantly changed the microbial community structure. In addition, the specificity of the microbial community was also changed by continuous cropping and was decreased after disease. Abundances of some microbial taxa associated with *P. notoginseng* also changed after continuous cropping. The results showed that continuous *P. notoginseng* cropping changed the soil microbial community and resulted in continuous cropping soil sickness. A large number of studies have shown that continuous cropping leads to the accumulation of organic and phenolic acids and affects the microbial community structure in rhizospheric soil (Blagodatskaya and Kuzyakov, 2013).

### Effects of Phenolic Acids on Soil Microorganisms

The number and species of rhizospheric soil microorganisms are important factors affecting plant growth, development and health (Berendsen et al., 2012; Yang et al., 2017). Phenolic acids exert direct effects on crops but can also indirectly affect microbial biomass, community structure and biodiversity (Qu and Wang, 2008; Zhou and Wu, 2012). Phenolic acids reduce the uniformity of fungi, which is beneficial to the enrichment of some fungi, but this is not beneficial for plants. Because most plant diseases are caused by fungi, the accumulation of rhizospheric fungi may exacerbate continuous cropping soil sickness (Zhang et al., 2020). Beta-diversity analysis showed that phenolic acids could significantly change bacterial community structure. VA in significantly reduced the bacterial Shannon index. Exogenous *p*-HA, FA, SA, and VA significantly increased the fungal Chao1 index and *p*-HA showed the most significant effect. There is growing evidence that plants can alter the soil microbiota by secreting bioactive molecules into the rhizosphere and that the microbiome can also be modified by root exudates to form host-specific microbial communities (Luo et al., 2020). VA affected bacterial specificity, and *p*-HA affected fungal specificity. Thus, VA and *p*-HA affect both microbial diversity and microbial specificity, and it is a matter of time until a specific community that is dependent on these two phenolic acids will form. Phenolic acids can change the soil microbial community structure and cause negative feedback between plants and microorganisms, which can cause toxic effects on host plants and aggravate continuous cropping obstacles. Phenolic acids can change soil microbial community structure and cause soil microbial imbalance, resulting in toxic effects on host plants, thus inducing continuous cropping soil sickness (Zhou et al., 2018; Jin et al., 2020).

The phenolic acids in *P. notoginseng* rhizospheric soil are clearly correlated with microbial community distribution (Yang et al., 2020). VA is positively correlated with most fungi and bacteria, indicating that VA can promote the growth of rhizospheric microbes (Wu et al., 2016). Para-HA was positively correlated with an increase in bacteria (*Lelliottia* and *Flavobacterium*) after *P. notoginseng* disease. This result indicates that *p*-HA was able to promote the growth of these two bacteria. The main sign of continuous cropping soil sickness in *P. notoginseng* is root rot, which is caused by fungi such as *Fusarium* and *Cylindrocarpon destructans* (Guo et al., 2009; Wen et al., 2020). FUNGuild also predicted that *Ilyonectria* was a plant pathogen. Para-HA was positively correlated with *Fusarium* and *Ilyonectria.* In conclusion, *p*-HA and VA are the main autotoxic phenolic acids in *P. notoginseng* continuous cropping.

### Soil Microorganisms Degrade Phenolic Acids

Previous studies have found that the rhizospheric environment and root exudates of plants interact with each other. On the one hand, changes in the rhizosphere affect the types and quantities of exudates; on the other hand, rhizosphere exudates also cause changes in the rhizosphere (Zhang et al., 2007). The half-life periods of *p*-HA, BA and *p*-CA were all less than 3 h, while the half-life of VA was less than 4.5 h. Thus, soil microorganisms can degrade phenolic acids rapidly. It is inferred that if the microbial community of *P. notoginseng* roots changes, the phenolic acid content of the *P. notoginseng* rhizospheric soil will increase or decrease accordingly.

### Suggestions for Solving *Panax notoginseng* Continuous Cropping Soil Sickness

Continuous cropping soil sickness in *P. notoginseng* urgently needs to be solved. Rhizospheric microecology involves a special interaction between crop-soil microorganisms and the environment. Crop root exudates and soil microorganisms interact in the soil, which jointly affects the growth and development of crops. Coordinating the relationships among these factors is the key to solving the problems associated with continuous cropping (Fu and Jia, 2012). This study creatively combined field investigation with laboratory experiments to determine the main phenolic acid autotoxins (*p*-HA and VA). Exogenously supplied phenolic acids were able to affect the diversity and abundance of soil microbes. There were strong correlations between phenolic acids and rhizospheric soil microorganisms. Soil microorganisms can also degrade phenolic acids quickly. It is a dynamic equilibrium. Therefore, maintaining a balance between phenolic acids and soil microorganisms can alleviate continuous cropping obstacles. Crop rotation and fallowing can also be used to balance the interaction between soil autotoxic substances and microorganisms before cultivation. Fallowing and crop rotation could maintain the dynamic balance between phenolic acids and soil microorganisms. Soil diseases can also be alleviated by adding beneficial microorganisms that can degrade phenolic acids to the soil.

## Data Availability Statement

The datasets presented in this study can be found in online repositories. The names of the repository/repositories and accession number(s) can be found below: https://www.ncbi.nlm.nih.gov/, PRJNA757903.

## Author Contributions

LB was responsible for experimental operation and manuscript writing. YL and JS were responsible for the collection of experimental samples. YD was responsible for solving technical problems. YT was responsible for the design of the experimental method and solving the problems encountered during the experiment. YW and FZ played a supervisory role. All authors contributed to the article and approved the submitted version.

## Conflict of Interest

The authors declare that the research was conducted in the absence of any commercial or financial relationships that could be construed as a potential conflict of interest.

## Publisher’s Note

All claims expressed in this article are solely those of the authors and do not necessarily represent those of their affiliated organizations, or those of the publisher, the editors and the reviewers. Any product that may be evaluated in this article, or claim that may be made by its manufacturer, is not guaranteed or endorsed by the publisher.
