# Supplementary material for: Interactions Between Phenolic Acids and Microorganisms in Rhizospheric Soil From Continuous Cropping of Panax notoginseng
Source: Front Microbiol. 2022 Feb 24;13:791603. doi: 10.3389/fmicb.2022.791603 (PMC8908257; doi:10.3389/fmicb.2022.791603)
Supplement: Supplementary file 1 [file Data_Sheet_1.docx]

**S. Table 1. Number and name of specific species of bacteria**

| Groups and quantity | Endemic species of bacteria |
| --- | --- |
| CK1 (9) | *Actinospica-robiniae-DSM-44927, Akkermansia-glycaniphila, Clostridiales-bacterium-CIEAF-012, Clostridium-leptum, Desulfovibrio-fairfieldensis, Exiguobacterium-indicum, Lactococcus-lactis, Pseudogulbenkiania-ferrooxidans-2002, Psychrobacter-marincola* |
| H-1Y (5) | *Acidobacteria-bacterium-LX128, Bacillus-sp-TAT112, Bdellovibrio-sp-oral-clone-CA006, Bifidobacterium-longum-subsp-longum,Tepidimicrobium-sp-GRC5* |
| H-2Y (6) | *Chitinophaga-rupis, Haloferula-sp-BvORR071, Leptolyngbya-cf-Albertano-Kovacik-green-Es-Yyy1800, Nocardioides-halotolerans, Pseudoxanthomonas-mexicana,Thermobispora-bispora* |
| H-3Y (4) | *Empedobacter-brevis, Flavobacterium-sp, Thermobacillus-composti, bacterium-Ellin515* |
| S-2Y (3) | *Bacillus-selenatarsenatis, Paenibacillus-hemerocallicola, Pedobacter-rhizosphaerae* |
| S-3Y (4) | *Candidatus-Metachlamydia-lacustris, Chthoniobacter-flavus, Lysobacter-ximonensis, Verrucomicrobium-sp-IMCC25902* |
| CK2 (4) | *Acidobacteria-bacterium-LX3, Garciella-sp-YIM-G1-1, Pirellula-sp, Proteus-mirabilis* |
| *p*-HA (4) | *Elizabethkingia-genomosp-4, Labilithrix-luteola,Lachnospiraceae-bacterium-28-4, Lactococcus-lactis* |
| BA (0) | *No endemic species* |
| FA (5) | *Bifidobacterium-longum-subsp-longum, Ectothiorhodospira-sp-enrichment-culture, Oscillibacter-sp-1-3, agricultural-soil-bacterium-SC-I-71, bacterium-Ellin515* |
| SA (5) | *Candidatus-Troglogloea-absoloni, Clostridium-pasteurianum-DSM-525-=-ATCC-6013, Fimbriimonas-ginsengisoli-Gsoil-348*, *Firmicutes-bacterium-JGI-0000119-C08, Psychrobacter-marincola* |
| VA (13) | *Acidobacteria-bacterium-WX72, Akkermansia-glycaniphila, Bacteroides-caecimuris, Chitinophaga-rupis, Clostridiales-bacterium-CIEAF-012 , Clostridium-leptum, Clostridium-perfringens-CPE-str-F4969，Clostridium-sp-ASF502, Desulfovibrio-fairfieldensis, Faecalibacterium-prausnitzii, Lachnospiraceae-bacterium-615, Parabacteroides-sp-YL27, Streptococcus-danieliae* |
| *p*-CA (5) | *Aquicella-siphonis, Myxococcus-fulvus,Streptomyces-sp-KP17, Turneriella-parva-DSM-21527, eubacterium-sp-22-7* |

**S. Table 2 Number and name of specific species of Fungal**

| groups and quantity | Fungal endemic species |
| --- | --- |
| CK1 (93) | *Acremonium-polychromum, Agonimia-allobata, Alternaria-dichondrae, Apiotrichum-laibachii, Arthrobotrys-oligospora, Aspergillus-chlamydosporus, Aspergillus-clavatonanicus, Aspergillus-westerdijkiae, Aureobasidium-thailandense, Bipolaris-cynodontis, Bipolaris-simmondsii, Candida-albicans, Candida-apicola, Catenulostroma-hermanusense, Cenococcum-geophilum, Cephalotrichum-stemonitis, Cetraspora-nodosa, Chaetosphaeria-myriocarpa, Chrysosporium-merdarium, Cladorrhinum-flexuosum, Claviceps-pusilla, Colletotrichum-gloeosporioides, Coniella-eucalyptorum, Conocybe-aurea, Coprinus-cordisporus, Corallomycetella-repens, Coriolopsis-aspera, Cyphellophora-gamsii, Cyphellophora-gamsii, Cyphellophora-sessilis, Cyrenella-elegans, Diutina-catenulata, Entoloma-graphitipes-f-cystidiatum, Exophiala-salmonis, Exserohilum-rostratum, Gonatophragmium-triuniae, Heterocephalacria-arrabidensis, Hirsutella-proturicola, Hyphodontia-microspora, Leptospora-rubella, Monocillium-indicum, Mortierella-capitata, Mucor-moelleri, Mucor-racemosus, Ochroconis-cordanae, Ochroconis-mirabilis, Ogataea-methanolica, Oidiodendron-truncatum, Ophiostoma-tsotsi, Papiliotrema-aurea, Paramyrothecium-humicola, Penicillium-adametzioides, Phialocephala-trigonospora, Phyllozyma-subbrunnea, Pilidium-concavum, Piskurozyma-taiwanensis, Pleurostoma-richardsiae, Podospora-cupiformis, Polyschema-sclerotigenum, Porostereum-spadiceum, Preussia-globosa, Rhodosporidiobolus-odoratus, Rhodotorula-paludigena, Roussoella-solani, Schizophyllum-commune, Schwanniomyces-occidentalis, Setophoma-chromolaenae, Slooffia-cresolica, Sphaerellopsis-filum, Sphaerellopsis-paraphysata, Spirosphaera-cupreorufescens, Talaromyces-helicus, Talaromyces-minioluteus, Talaromyces-rugulosus, Tausonia-pullulans, Tetracladium-furcatum, Thermomyces-dupontii, Thielavia-terrestris, Thielaviopsis-basicola, Tolypocladium-parasiticum, Tremella-fuciformis, Trichocladium-opacum, Veronaea-japonica, Vishniacozyma-taibaiensis, Vishniacozyma-tephrensis, Wallemia-canadensis.* |
| H-1Y (22) | *Achaetomium-luteum, Arthrographis-chlamydospora, Arxiella-dolichandrae, Aspergillus-deflectus, Byssochlamys-lagunculariae, Candida-intermedia, Conocybe-papillata, Coprinellus-curtus, Cystobasidium-slooffiae, Desmazierella-acicola, Hyphodontia-crustosa, Muscodor-suthepensis, Neodevriesia-poagena, Neoidriella-desertorum, Penicillium-sumatraense, Phlebia-acerina, Rhizophagus-clarus, Rhizophagus-custos, Rhizopus-arrhizus,Sonoraphlyctis-ranzonii, Ustilago-trichophora, Veronaea-compacta* |
| H-2Y (31) | *Absidia-repens, Agaricus-bisporus, Antennariella-placitae, Archaeospora-schenckii, Arthrobotrys-musiformis, Arthrophiala-arthrospora, Auxarthron-alboluteum,Barnettozyma-californica, Coniochaeta-decumbens, Coprinopsis-clastophylla, Dissoconium-eucalypti, Geastrum-triplex, Gibellulopsis-chrysanthemi, Hannaella-sinensis, Hirsutella-subulata, Hypomyces-ochraceus, Hypoxylon-carneum, Leohumicola-minima, Lophiotrema-eburnoides, Metarhizium-rileyi, Microdochium-trichocladiopsis, Mycena-sanguinolenta, Neophaeococcomyces-catenatus, Phallus-rugulosus, Podospora-intestinacea, Psathyrella-sulcatotuberculosa, Radulomyces-paumanokensis, Rhizophagus-intraradices, Scedosporium-prolificans, Strobilomyces-strobilaceus, Trechispora-invisitata* |
| H-3Y (16) | *Acremoniopsis-suttonii, Annulohypoxylon-stygium, Arthrobotrys-amerospora, Athelopsis-lembospora, Cunninghamella-elegans, Helicoma-fumosum, Hyalorbilia-juliae, Hyphodontia-arguta, Lecanicillium-fusisporum, Leucocoprinus-birnbaumii, Leucocoprinus-ianthinus, Myceliophthora-fergusii, Pleuroascus-nicholsonii,Thelonectria-diademata, Tomentella-papuae, Udeniomyces-pyricola* |
| S-2Y (11) | *Archaeospora-trappei, Coprinopsis-phlyctidospora, Duddingtonia-flagrans, Erythrobasidium-hasegawianum, Magnaporthe-grisea, Myrmecridium-thailandicum, Myrothecium-inundatum, Myrothecium-inundatum, Phanerochaete-bambusicola, Ramophialophora-humicola, Volutella-consors, Xylaria-acuminatilongissima* |
| S-3Y (10) | *Ampelomyces-quisqualis, Bjerkandera-adusta, Dothiorella-rosulata, Mortierella-oligospora, Nemania-diffusa, Piskurozyma-cylindrica, Talaromyces-radicus, Terana-coerulea, Torula-masonii, Xylaria-venosula* |
| CK2 (12) | *Clavatospora-longibrachiata, Coniochaeta-decumbens, Helicogloea-farinacea, Malassezia-sympodialis, Metarhizium-lepidiotae, Mucor-racemosus, Periconia-echinochloae, Psilocybe-inquilina, Ramicandelaber-taiwanensis, Rhexodenticula-cylindrospora, Rhizosphaera-oudemansii, Tomentella-stuposa* |
| *p*-HA (22) | *Barnettozyma-californica, Coprinopsis-urticicola, Coprinus-annuloporus, Ctenomyces-serratus, Cystobasidium-slooffiae, Dictyosporium-heptasporum, Gibberella-tricincta, Gongronella-koreana, Hymenoscyphus-menthae, Hypoxylon-monticulosum, Lipomyces-kononenkoae, Minimelanolocus-asiaticus, Nemania-diffusa, Pholiota-microspora, Rhizopus-arrhizus, Rhytidhysteron-rufulum, Saturnispora-diversa, Schizopora-ovispora, Setophaeosphaeria-hemerocallidis, Stachybotrys-chartarum, Starmerella-bacillaris, Sterigmatomyces-halophilus* |
| BA (14) | *Absidia-repens, Biatriospora-mackinnonii, Fusarium-redolens, Golubevia-pallescens, Hamigera-paravellanea, Itersonilia-perplexans, Leohumicola-minima, Leucocoprinus-jubilaei, Palmiascoma-gregariascomum, Panaeolus-papilionaceus, Penicillium-sumatraense, Stemphylium-sp, Sydowia-polyspora, Thermomyces-dupontii* |
| FA (9) | *Alternaria-dichondrae, Annulohypoxylon-stygium, Cenococcum-geophilum, Daldinia-caldariorum, Exophiala-sideris, Hannaella-oryzae, Paramyrothecium-tellicola, Pleurotus-pulmonarius, Stereum-sanguinolentum* |
| SA (15) | *Agaricus-abruptibulbus, Anthracobia-melaloma, Byssochlamys-lagunculariae, Candida-intermedia, Cephalotrichiella-penicillata, Coniochaeta-fasciculata, Conocybe-aurea, Graphiopsis-chlorocephala, Hyalorbilia-juliae, Hyphoderma-subsetigerum, Ochroconis-cordanae, Phialophora-livistonae, Spizellomyces-pseudodichotomus, Thelonectria-mammoidea, Verticillium-zaregamsianum* |
| VA (10) | *Acremonium-hennebertii, Colletotrichum-aciculare, Helicoma-isiola, Leohumicola-verrucosa, Leucocoprinus-cretaceus, Mycena-pura, Rhizopus-microsporus, Talaromyces-helicus, Tomentella-punicea, Tulostoma-squamosum* |
| *p*-CA (12) | *Amanita-orientigemmata, Cutaneotrichosporon-moniliiforme, Exophiala-moniliae, Lentinus-squarrosulus, Mortierella-oligospora, Oidiodendron-cereale, Penicillium-oxalicum, Scytalidium-album, Steccherinum-bourdotii, Tetraplosphaeria-nagasakiensis* |
